# Association of Posttransplant Kidney Function With Patient Reported Outcomes: A Single Center's Experience Over Nearly Two Decades

**DOI:** 10.1111/ctr.70500

**Published:** 2026-03-02

**Authors:** Peter Thorne, Laura A. Binari, Scott A. Rega, Guneet Kochar, Rachel C. Forbes, C. Wright Pinson, Irene D. Feurer, Beatrice P. Concepcion

**Affiliations:** ^1^ Division of Nephrology and Hypertension University of Minnesota Medical Center Minneapolis Minnesota USA; ^2^ Division of Nephrology and Hypertension Vanderbilt University Medical Center Nashville Tennessee USA; ^3^ Vanderbilt Transplant Center Nashville Tennessee USA; ^4^ Metrolina Nephrology Associates, P.A. Charlotte North Carolina USA; ^5^ Division of Kidney and Pancreas Transplantation Vanderbilt University Medical Center Nashville Tennessee USA; ^6^ Departments of Surgery and Biostatistics Vanderbilt University Medical Center Nashville Tennessee USA; ^7^ Section of Nephrology University of Chicago Chicago Illinois USA

## Abstract

**Background:**

Impaired kidney function in the non‐transplant chronic kidney disease (CKD) population has been shown to negatively affect patients’ health related quality of life (HRQOL). The relationship between posttransplant graft function, as measured by estimated glomerular filtration rate (eGFR), and patient‐reported outcomes (PRO) remains poorly understood. This study evaluates the associations between eGFR and PRO in kidney transplant recipients to inform clinical strategies aimed at optimizing both physical and psychological well‐being.

**Methods:**

Longitudinal data were collected using previously‐described procedures and a multi‐survey PRO battery. Logistic regression models evaluated relationships, at the last follow‐up point, between eGFR strata, time posttransplant, age at PRO, whether there had been a previous kidney transplant or the donor was deceased or living, and the likelihood of physical or mental HRQOL being substantive low and of symptoms of depression or anxiety being reported. Parallel multivariable mixed effects models, that included all longitudinal data points for each participant, examined relationships between eGFR and continuous PRO scores and their temporal trajectories.

**Results:**

The study included 2116 adult kidney transplant recipients and over 9500 unique multi‐survey observation points over a 19‐year period. After adjusting for age (*p* < 0.001), donor type, time posttransplant, and prior kidney transplantation, there was a statistically significant association between eGFR/CKD strata and the likelihood of physical HRQOL being substantively low (*p* < 0.001) at the last PRO assessment. CKD stage 4 or 5 was independently associated with a 1.5 times increased likelihood of reporting symptoms of depression (OR: 1.50; 95% CI 1.16, 1.95) and anxiety (OR = 1.48; 95%CI: 1.14, 1.92) compared to those with eGFR ≥ 60 mL/min/1.73m^2^. Longitudinal analyses comprising all data points demonstrated that increased eGFR was associated with better physical and mental HRQOL and reduced symptoms of depression and anxiety.

**Conclusions:**

Impaired graft function is significantly associated with decreased physical HRQOL and increased symptoms of depression and anxiety in kidney transplant recipients. These findings underscore the importance of close monitoring and early interventions targeting physical and psychological well‐being as graft function declines.

## 1 Introduction

Patients with end stage kidney disease are often treated with dialysis or kidney transplantation, with the latter considered superior because kidney transplantation has a survival advantage compared to dialysis [1]. Advances in the care of kidney transplant recipients have led to notably improved outcomes. This prolonged survival has led to an increased focus on patient reported outcomes (PROs) as part of evaluating treatment effectiveness [2]. Patient‐reported outcomes (PRO), such as health‐related quality of life (HRQOL), may be assessed using various standardized tools that measure multidimensional constructs reflecting social, mental, and physical health [3]. Evaluation of PROs allows for the quantification of disease consequences from the patient's perspective. Enhancing or maintaining a patient's HRQOL and addressing patient‐reported symptoms of psychiatric comorbidities such as depression and anxiety are important aspects of the care of any chronic health condition such as chronic kidney disease (CKD) or kidney transplantation.

Despite advances in the care of kidney transplant recipients, loss of graft function and graft failure still occur. Impaired kidney function has been shown to negatively affect patients’ HRQOL in the non‐transplant CKD population, particularly in the physical domains, and CKD is associated with increased patient‐reported symptoms of depression and anxiety [4, 5, 6, 7, 8]. This association is likely multifactorial and related to the accumulation of comorbidities, physiologic changes from kidney impairment such as anemia, fatigue, and chronic inflammation. In addition, increased exposure to medical interventions such as increased medication burden, clinic visits, and hospitalizations likely plays a role. There is a need for further data examining the association between graft function and PROs after kidney transplantation.

To this end, the primary aim of the present study was to evaluate associations between graft function, as measured by estimated glomerular filtration rate (eGFR), and the likelihood of persons reporting impaired physical or mental HRQOL and/or the presence of symptoms of depression and anxiety after kidney transplantation. The secondary aim was to evaluate, using longitudinal data, contemporaneous associations between graft function and these PROs and the temporal trajectories of these measures in a large cohort of kidney transplant recipients. We hypothesized that impaired posttransplant kidney function, as reflected by the severity of kidney dysfunction as measured by CKD stage based on eGFR (denoted as stages 3a, 3b, 4, 5), would be associated with increased risk for clinically substantive reductions in physical and mental HRQOL and increased risk for symptoms of depression and anxiety. Knowledge of contributing factors associated with reduced PROs after kidney transplantation can help increase vigilance and guide earlier interventions by clinicians.

## 2 Methods

### 2.1 Study Design

Longitudinal PRO data were collected in adult kidney transplant recipients under an IRB‐approved protocol using a previously described survey battery and a rolling enrollment process that maximizes the number of participants and follow‐up duration [9]. A rolling enrollment process results in persons being able to participate before or after transplantation regardless of previous participation. Standard posttransplant points are one, three, six months and annually. Inclusion criteria were adult kidney transplant recipients who completed at least one posttransplant multi‐survey PRO data point. Patient characteristics included age (years) at each measurement point, gender, race/ethnicity, donor type (deceased or living), whether there had been a previous kidney transplant, and longitudinal posttransplant serum creatinine (mg/dl). eGFR (ml/min/1.73m^2^) was calculated from serum creatinine, age, and gender using the CKD‐EPI 2021 equation at each PRO datapoint [10]. Continuous eGFR data were stratified utilizing nomenclature for CKD staging measured by glomerular filtration rate: 1) Normal/CKD Stage 1 or 2 (≥ 60 mL/min/1.73m^2^); 2) CKD Stage 3a (45–59 mL/min/1.73m^2^); 3) CKD Stage 3b (30–44 mL/min/1.73m^2^); and 4) CKD Stage 4 or 5 (≤ 29 mL/min/1.73m^2^). Time posttransplant was computed (in months) at each PRO data point and indexed to the date of the most recent or only kidney transplant.

### 2.2 PRO Measures

Health‐related quality of life was measured using version 1 of the SF‐36 Health Survey [11], which is widely used in kidney transplant research [12]. The SF‐36 physical and mental component summary scores (PCS and MCS) were computed from eight differentially weighted scales, and interpreted in relation to the general population standardized mean of 50 ± 10, with the “normal” range considered to be 40 to 60. Higher scores represent better HRQOL. In addition, PCS and MCS scores were classified using the convention of a minimally important difference for these measures being a half standard deviation, as low (< 35, more than a half standard deviation below the general population) or not low (≥ 35) [13].

The Center for Epidemiologic Studies Depression Scale (CES‐D) was used to measure self‐reported symptoms of depression [14]. Total scores may range from 0 to 60 reflecting symptoms experienced over the past week, with higher scores representing more severe symptoms of depression. Total CES‐D scores were interpreted as: 0–9 (none), 10–16 (mild), 17–24 (moderate), and ≥ 25 (moderate to severe) (ref Radloff) and then dichotomized to reflect the presence (total score ≥ 10) or absence of symptoms of depression (total score < 10). Symptoms of anxiety were reported using the Beck Anxiety Inventory (BAI) [15]. Total scores may range 0 to 63. Higher scores represent greater symptom severity and are interpreted as: 0–7 (minimal), 8–15 (mild), 16–25 (moderate), and ≥ 26 (severe) [15]. Total BAI scores were subsequently dichotomized to reflect the presence of symptoms that were mild to severe (total score ≥ 8) versus the absence of or minimal symptoms of anxiety (total score < 8).

### 2.3 Data Management and Statistical Methods

Data were analyzed using summary statistics. Multivariable logistic regression models evaluated relationships at each participant's last PRO measurement point, between eGFR strata (ref: ≥ 60 mL/min), time posttransplant (months), age at the PRO assessment (years), whether there had been a previous kidney transplant (ref: single transplant), whether the donor was deceased (ref: living), and the likelihood of PCS and MCS being low (score < 35) and symptoms of depression and anxiety being present. Parallel multivariable mixed effects models, which included the same set of covariables, a random intercept at the person‐level, and all available longitudinal data points, examined relationships between eGFR (expressed as ml/min/1.73 m^2^/10) and continuous PCS, MCS, CES‐D, and BAI scores. The multivariable logistic regression and mixed effects models were developed for the purpose of identifying factors associated with PROs and model‐specific predictive performance metrics are not reported. Some data were managed using Research Electronic Data Capture (REDCap) tools hosted at Vanderbilt University Medical Center and data were analyzed using IBM SPSS Statistics (v 28, Armonk, NY) [16, 17].

The clinical and research activities being reported are consistent with the Principles of the Declaration of Istanbul as outlined in the Declaration of Istanbul on Organ Trafficking and Transplant Tourism. The study was approved by the Vanderbilt University Institutional Review Board.

## 3 Results

This study included 2116 kidney transplant recipients and over 9500 unique multi‐survey PRO observation points reported over a 19‐year period (between 2002 and 2020) and a median follow‐up of five years from transplant to the last PRO datapoint. The number of posttransplant survey observation points per person ranged from 1 (*n* = 316, 15% of participants) to 15 (*n* = 1 participant), with a median of 4 and a 75^th^ percentile of 6 observations. Among the 1800 participants with two or more observation points, the average time between the initial and final posttransplant observations was 4.9 ± 3.8 years. Summary participant data are outlined in Table 1.

**TABLE 1:** Patient characteristics.

(*N* = 2116)	
Age at first/only PRO data point (years)	50 (13)
Gender	
Female	890 (42)
Male	1226 (58)
Race/ethnicity	
White	1432 (68)
Black	605 (29)
Other	79 (4)
Donor type	
Deceased	1311 (62)
Living	805 (38)
Previous kidney transplant	
No	2000 (95)
Yes	116 (6)
eGFR at last PRO observation point (ml/min/1.73m^2^)	54 ± 24
eGFR stratum at last PRO observation point	
Normal/CKD Stage 1 or 2 (≥ 60)	878 (42)
CKD Stage 3a (45–59)	518 (24)
CKD Stage 3b (30–44)	365 (17)
CKD Stage 4 or 5 (≤ 29)	355 (17)
Dates of first/only kidney transplant	02/11/1988 to 01/30/2019
Time posttransplant at last PRO observation (months)	60 (29, 105)

*Note:* Table entries are mean ± SD, frequency (%), or median (interquartile thresholds).

### 3.1 Physical HRQOL

Multivariable logistic regression indicated that, after adjusting for age (*p* < 0.001), donor type, time posttransplant at the last PRO assessment, and prior kidney transplantation, there was a statistically significant association (*p* < 0.001) between eGFR/CKD strata and the likelihood of physical HRQOL being substantively low (Table 2, panel A). Specifically, persons with CKD stage 3b were about 1.4 times more likely (OR = 1.38; 95%CI 1.04, 1.83) and those with CKD stage 4 or 5 were about twice as likely (OR = 2.05; 95%CI 1.55, 2.71) to have substantively low physical quality of life compared to those with eGFR ≥ 60 mL/min/1.73m^2^. The overlapping confidence intervals for these effects indicate that, compared to those with eGFR ≥ 60 mL/min/1.73m^2^, model‐adjusted likelihood of substantively reduced PCS was increased when posttransplant eGFR was less than 45 mL/min/1.73m^2^ and the risk associated with CKD stage 3b and CKD stage 4 or 5 was not ordinal. The parallel mixed effects model, which included 9502 observations in 2116 persons (Table 3, Panel A), indicated that PCS scores were stable over time (effect of time *p* = 0.93) and that, after adjusting for the negative effect of increased age (*p* < 0.001) on PCS, increased eGFR was associated with better physical HRQOL.

**TABLE 2:** Logistic regression models of likelihood of whether diminished physical and mental quality of life and symptoms of depression and anxiety at last assessment.

	A. Low physical quality of life *n* = 2116 persons			B. Low mental quality of life *n* = 2116 persons			C. Any symptoms of depression *n* = 2051 persons			D. Any symptoms of anxiety *n* = 2,062 persons		
	Odds ratio	95% CI	*p*‐value	Odds ratio	95% CI	*p*‐value	Odds ratio	95% CI	*p*‐value	Odds ratio	95% CI	*p*‐value
Repeat transplant (ref: single transplant)	0.78	0.49, 1.26	0.32	1.39	0.83, 2.33	0.21	1.10	0.75, 1.61	0.64	1.14	0.77, 1.68	0.51
Deceased donor (ref: living donor)	0.99	0.80, 1.23	0.93	1.02	0.78, 1.35	0.87	1.20	1.00, 1.45	0.06	0.99	0.82, 1.20	0.95
Age (years)	1.02	1.02, 1.03	<0.001	0.99	0.98, 1.00	0.17	1.00	0.99, 1.01	0.85	1.00	1.00, 1.01	0.48
Time from transplant (months)	1.00	1.00, 1.00	0.71	1.00	1.00, 1.00	0.19	1.00	1.00, 1.00	0.90	1.00	1.00, 1.00	0.15
eGFR Stratum (ml/min/1.73m^2^)			<0.001			0.55			0.002			0.02
45–59 (ref: ≥60)	1.03	0.79, 1.34	0.85	0.87	0.62, 1.24	0.45	0.88	0.70, 1.10	0.25	0.99	0.79, 1.24	0.91
30–44 (ref: ≥60)	1.38	1.04, 1.83	0.03	1.17	0.82, 1.69	0.40	1.17	0.91, 1.51	0.22	1.14	0.88, 1.47	0.32
≤ 29 (ref: ≥60)	2.05	1.55, 2.71	<0.001	1.08	0.74, 1.57	0.72	1.50	1.16, 1.95	0.002	1.48	1.14, 1.92	0.003
Constant	0.08		<0.001	0.17		<0.001	0.60		0.01	0.56		0.005

**TABLE 3:** Longitudinal mixed effects models of posttransplant physical and mental quality of life and symptoms of depression and anxiety.

	A. Physical quality of life *n* = 2116 persons *n* = 9502 observations			B. Mental quality of life *n* = 2116 persons *n* = 9502 observations			C. Symptoms of depression *n* = 2051 persons *n* = 8170 observations			D. Symptoms of anxiety *n* = 2062 persons *n* = 8367 observations		
	Beta	95% CI	*p*‐value	Beta	95% CI	*p*‐value	Beta	95% CI	*p*‐value	Beta	95% CI	*p*‐value
Repeat transplant (ref: single transplant)	−0.19	−1.57, 1.19	0.78	−0.54	−1.87, 0.80	0.43	0.40	−0.79, 1.60	0.51	−0.04	−1.06, 0.99	0.95
Deceased donor (ref: living donor)	−0.20	−0.91, 0.51	0.58	−0.51	−1.19, 0.16	0.14	1.15	0.55, 1.76	<0.001	0.39	−0.14, 0.91	0.15
Age (years)	−0.17	−0.19, −0.14	<0.001	0.02	−0.00, 0.05	0.08	−0.01	−0.03, 0.02	0.62	0.01	−0.01, 0.03	0.40
Time from transplant (months)	2.28 × 10^−4^	−0.00, 0.01	0.93	−0.01	−0.01, −0.00	0.001	0.01	0.00, 0.01	<0.001	0.00	−0.00, 0.00	0.75
eGFR (ml/min/1.73m^2^)	0.43	0.31, 0.55	<0.001	0.15	0.03, 0.27	0.02	−0.17	−0.27, −0.06	0.003	−0.18	−0.27, −0.09	<0.001
Intercept	49.56	47.48, 51.64	<0.001	48.74	46.75, 50.74	<0.001	10.20	8.42, 11.98	<0.001	8.12	6.58, 9.66	<0.001

### 3.2 Mental HRQOL

The logistic regression model demonstrated that there were no significant associations between any covariables, including eGFR strata, and the likelihood of MCS being substantively low (Table 2, Panel B). The parallel mixed effects model indicated that PCS scores declined over time (*p* = 0.001) and that higher eGFR was associated with better mental HRQOL (*p* = 0.02) (Table 3, Panel B).

### 3.3 Symptoms of Depression

The logistic regression model demonstrated that CKD stage 4 or 5 was independently associated with an overall 1.5 times (OR: 1.50; 95% CI 1.16, 1.95) increased likelihood of symptoms of depression compared to those with eGFR ≥ 60 mL/min/1.73m^2^ (Table 2, Panel C). The mixed effects model, which included 8170 observations in 2051 persons, demonstrated that symptoms of depression increased over time, were higher in recipients of deceased donor kidney transplants, and that increased eGFR was associated with reduced patient‐reported symptoms of depression (all *p* < 0.004) (Table 3, Panel C).

### 3.4 Symptoms of Anxiety

Multivariable logistic regression demonstrated that, similar to the analysis of likelihood of symptoms of depression, CKD stage 4 or 5 was associated with an overall 1.5 times (OR = 1.48; 95%CI: 1.14, 1.92) increased likelihood of reporting symptoms of anxiety compared to those with eGFR ≥ 60 mL/min/1.73m^2^ (Table 2, Panel D). The parallel mixed effects model, which included 8367 observations in 2062 persons, was consistent with the logistic regression model and demonstrated that patient‐reported symptoms of anxiety were stable posttransplant (effect of time *p* = 0.15) and that increased eGFR was associated with reduced symptoms of anxiety (*p* < 0.001) (Table 3, Panel D).

## 4 Discussion

The results of this study demonstrate a statistically significant and clinically relevant decrease in patient reported physical HRQOL and increased patient‐reported symptoms of depression and anxiety in a vulnerable posttransplant population. When compared to kidney transplant recipients with eGFR ≥ 60 mL/min/1.73m^2^ (normal kidney function or CKD stage 1 or 2), those with CKD stage 4 or 5 and those with CKD stage 3b were twice as likely and 1.4 times more likely, respectively, to have substantively reduced physical HRQOL. In addition, kidney transplant recipients with CKD stage 4 or 5 were about 1.5 times more likely to report symptoms of depression and anxiety compared to patients with eGFR ≥ 60 mL/min/1.73m^2^. Parallel, longitudinal multivariable models supported these findings and demonstrated that decreased eGFR was associated with worse physical HRQOL and increased symptoms of depression and anxiety on self‐report inventories.

Our findings are consistent with studies that have shown a trend of decreased PCS as CKD advances in the general CKD population [18]. We suspect the finding of reduced PCS in our posttransplant population with CKD stage 4 or 5 are multifactorial with some contributing factors being similar to the general CKD population and some being unique to the posttransplant population. Higher disease burden as CKD progresses can include increased symptoms from additional comorbidities, physiologic changes from kidney impairment like worsening anemia, and increased cardiovascular complications. In addition, kidney transplant recipients are often exposed to additional interventions such as those needed to treat rejection episodes or opportunistic infections from immunosuppression which can contribute to increased need for hospitalization and contribute to deconditioning and a sense of reduced physical wellness.

There was not a statistically significant association between worsening kidney function and MCS < 35. When each of the CKD stages 3–5 were compared to normal kidney function and CKD stage 1 or 2, there was a trend towards having a higher chance of an MCS < 35 but none of these findings reached statistical significance. This is in line with previous studies that have shown no difference in MCS between pretransplant waitlisted patients with ESKD and kidney transplant recipients [19]. In addition, it should be noted that posttransplant patients and especially kidney transplant recipients, typically have high MCS at baseline so it is not especially surprising that there was no statistically significant difference in this group [20].

The increased patient‐reported symptoms of depression and anxiety are also likely multifactorial. We hypothesize that many of the same factors contributing to the lower PCS noted above also contribute to increased anxiety and depression symptoms. Increased medical interventions, fatigue, and accumulation of additional comorbidities can all contribute to a decreased sense of well‐being and increased anxiety or depression. Concerns about graft loss as CKD progresses and subsequent loss of independence, higher pill burden, fear of return to dialysis, or fear of need for re‐transplantation could certainly also be playing a role. Regardless of the underlying cause, it remains critical that health care providers monitor for these symptoms in this patient population and provide appropriate support and treatment.

Previous studies examining PROs in kidney transplant recipients have shown mixed results. Generally, kidney transplant recipients have been shown to have improved PROs compared to transplant candidates and patients on dialysis while other studies have shown that kidney transplant recipients still have lower PRO measures when compared to the general population [3, 21]. The present study included a significantly larger cohort than that used in many of these previous similar studies and adds to the available literature around PROs in the post kidney transplant population.

Our results build on those presented in similar studies. For instance, Neri et al. demonstrated in a cross‐sectional study of 577 kidney transplant recipients that several HRQOL dimensions were diminished as posttransplant kidney function declines [22]. This study utilized the KDQOL‐36, Euro‐QoL 5, and Health Utility Index Mark III to evaluate patient‐reported HRQOL. They found a statistically and clinically significant decrease in PCS as eGFR declined and no significant difference in MCS. They did not use the CES‐D or BAI in their study. These findings and ours are aligned with other studies which examined the CKD population prior to transplant that generally showed a reduced HRQOL as summarized in a systematic review by Alhaji et al. [4].

Our results differ from some previous studies. Lippe et al. performed a 5‐year prospective longitudinal study on self‐reported quality of life in 110 patients who transitioned from dialysis to kidney transplant [19]. This group measured HRQOL using the KDQOL‐SF, SF‐36, and found no clinically significant change in SF‐36 HRQOL posttransplant between dialysis patients and kidney transplant recipients. Kidney transplant recipients also had highly significant decreases in HRQOL when compared to the general population except in the domains of bodily pain and mental health. They did not specifically look at the CES‐D or BAI to evaluate depression/anxiety in this population and interestingly when they stratified patients by eGFR they did not see any difference in HRQOL. This may have been in part due to the shorter median follow‐up time posttransplant in this study compared to ours (41 months vs 74 months).

The strengths of this study include the very large number of individual patients included in our analysis and the number of individual observations included in the longitudinal multivariable mixed effects models. We were able to adjust for several characteristics including age, time posttransplant, donor type, and prior transplantation. Our study is further strengthened by the use of multiple patient‐reported outcome tools to evaluate multiple domains of HRQOL, depression, and anxiety in this patient population. To our knowledge we are the first to report PRO data on a kidney transplant population of this size with longitudinal data regarding multiple validated tools examining anxiety/depression and PCS/MCS scores in relation to CKD stage posttransplant.

Our study does present some limitations. Our study is limited by its single‐center design and the ability to generalize our data, although we have a large number of individual patients and our center performs transplants for patients from a large geographical catchment area which should help to offset this limitation. Like other studies with a similar design, we can only provide associations and not evidence of causality. Detailed analysis of potential thresholds for rates of change in eGFR and their relation to PRO was not performed in this cohort that had heterogeneous numbers and temporal cadences of follow‐up points but is an area of future research.

Our data add to the growing number of studies evaluating PROs in the kidney transplant population. This study provides strong evidence in support of the need for close monitoring and additional support of posttransplant patients as kidney function declines and the importance of maintaining graft function posttransplant to improve PROs. Potential interventions targeting physical and mental health for this patient population include additional psychosocial support or interventions, increased frequency of patient monitoring, physical therapy or upstream support prior to patients progressing to CKD stage 4 or 5. These interventions are a vital and often overlooked component of the care of these patients. Anxiety and depression predict risk of sarcopenia in patients with CKD and treatment may mitigate this risk [23]. In addition, Ferreira and colleagues performed a systematic review and meta‐analysis that demonstrated exercise interventions improve depression and anxiety in patients with CKD [24]. Early referral to physical therapy or an exercise program in the transplant population as CKD progresses may show a dual benefit of improvement in anxiety and depression along with improvement in PCS. Increased awareness so these interventions are used earlier in this patient population may improve outcomes. Future studies are needed to further examine these potential interventions and to evaluate longitudinal impacts on PROs in the kidney transplant population.

In conclusion, this large study of kidney transplant recipients demonstrated a statistically significant decline in physical HRQOL in CKD stage 3b, 4, and 5 compared to patients with eGFR ≥ 60 mL/min/1.73m^2^, and increased patient‐reported symptoms of depression and anxiety in CKD stage 4 or 5 when compared to patients with eGFR ≥ 60 mL/min/1.73m^2^. These data suggest the importance for clinicians to monitor this vulnerable patient population closely and to consider the need for additional physical and psychosocial support as graft function declines.

## Author Contributions

Peter Thorne: acquisition of data, drafting the article, final approval of the version to be published, agreement to be accountable for all aspects of the work pre‐ and post‐publication; Laura A. Binari, Guneet Kochar, Rachel C. Forbes: acquisition of data, critical revision of the article for important intellectual content, final approval of the version to be published, agreement to be accountable for all aspects of the work pre‐ and post‐publication; Scott A. Rega: acquisition of data, analysis and interpretation of data, critical revision of the article for important intellectual content, final approval of the version to be published, agreement to be accountable for all aspects of the work pre‐ and post‐publication; C. Wright Pinson: conception and design, critical revision of the article for important intellectual content, final approval of the version to be published, agreement to be accountable for all aspects of the work pre‐ and post‐publication; Irene D. Feurer and Beatrice P. Concepcion: conception and design, drafting the article, acquisition of data, analysis and interpretation of data, final approval of the version to be published, agreement to be accountable for all aspects of the work pre‐ and post‐publication

## Funding

The project described was supported by CTSA award No. UL1TR000445 from the National Center for Advancing Translational Sciences. Its contents are solely the responsibility of the authors and do not necessarily represent official views of the National Center for Advancing Translational Sciences or the National Institutes of Health.

## Disclosures

The authors have nothing to report

## Conflicts of Interest

The authors declare no conflicts of interest.

## Data Availability

Data is available from the authors upon request. The data that support the findings of this study are available from the corresponding author upon reasonable request.
